# Molecular mechanisms of olfactory detection in insects: beyond receptors

**DOI:** 10.1098/rsob.200252

**Published:** 2020-10-07

**Authors:** Hayden R. Schmidt, Richard Benton

**Affiliations:** Center for Integrative Genomics, Faculty of Biology and Medicine, University of Lausanne, CH-1015, Lausanne, Switzerland

**Keywords:** olfaction, receptor, signalling, *Drosophila*, physiology, neuron

## Abstract

Insects thrive in diverse ecological niches in large part because of their highly sophisticated olfactory systems. Over the last two decades, a major focus in the study of insect olfaction has been on the role of olfactory receptors in mediating neuronal responses to environmental chemicals. *In vivo*, these receptors operate in specialized structures, called sensilla, which comprise neurons and non-neuronal support cells, extracellular lymph fluid and a precisely shaped cuticle. While sensilla are inherent to odour sensing in insects, we are only just beginning to understand their construction and function. Here, we review recent work that illuminates how odour-evoked neuronal activity is impacted by sensillar morphology, lymph fluid biochemistry, accessory signalling molecules in neurons and the physiological crosstalk between sensillar cells. These advances reveal multi-layered molecular and cellular mechanisms that determine the selectivity, sensitivity and dynamic modulation of odour-evoked responses in insects.

## 1. Introduction

Insects are one of the most successful classes of eukaryotes on Earth, making up approximately half of all terrestrial species [1]. They occupy an incredibly diverse range of habitats, encompassing tropical forests, deserts and the extremes of the polar regions. Many species exert an important influence on human health through their roles as disease vectors [2], crop pollinators [3] and agricultural pests [4]. The ecological adaptability of insects relies, in part, on their sophisticated olfactory systems, which allow detection and responses to innumerable volatile signals in the environment.

Studies of anatomical, physiological and behavioural aspects of insect olfaction have a long history in the twentieth century, using diverse model species [5]. Over the last two decades, there has been a particular focus on identifying and functionally characterizing olfactory receptors [6–8], as well as the neuronal circuits in which they are expressed and the odour-driven behaviours they control [9,10], notably in *Drosophila melanogaster*. There are two main classes of insect olfactory receptors: odorant receptors (ORs) [11,12] and ionotropic receptors (IRs) [13]. ORs are a family of seven-pass transmembrane ion channels, while IRs are three-pass transmembrane proteins distantly related to synaptic ionotropic glutamate receptors (iGluRs) [6–8,14]. Most olfactory sensory neurons (OSNs) express two different ORs or IRs: a unique ‘tuning' receptor that recognizes a set of ligands or odorants, and a co-receptor (ORCO for ORs, and either IR8a or IR25a for IRs). These co-receptors are not known to recognize any naturally occurring ligands, but form heteromeric complexes with tuning receptors to enable sensory cilia targeting and signalling [15–17].

**Figure 1.**
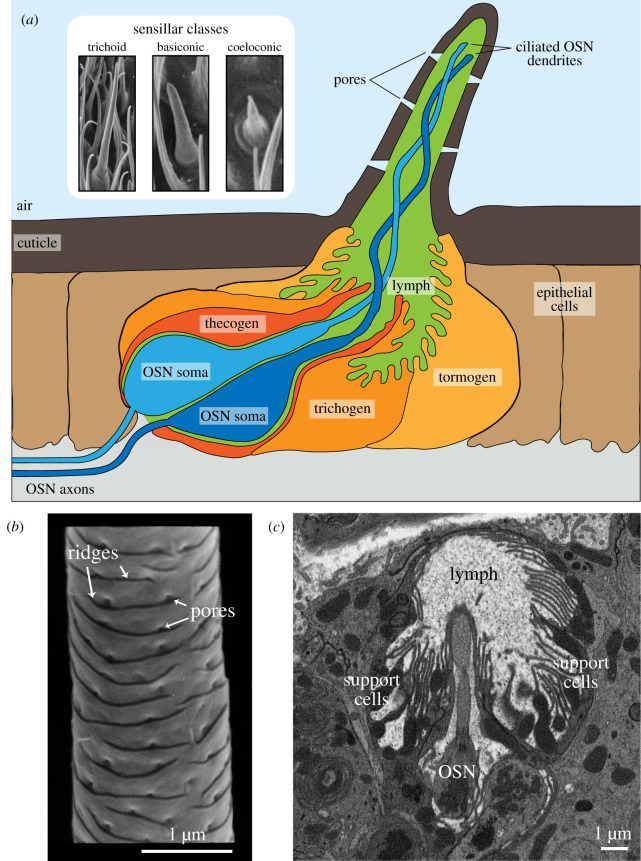
Insect olfactory sensillar morphology. (*a*) Schematic representation of an olfactory sensillum (see text for details). Inset: representative electron microscopy images of the main morphological classes of olfactory sensilla, here from *D. melanogaster* antennae (adapted from [18]). (*b*) Electron microscopy image of a trichoid sensillum from *B. mori* [19]. (*c*) Electron microscopy image of a *D. melanogaster* trichoid sensillum (at4) prepared using the CryoChem method and imaged using *en bloc* heavy metal staining (adapted from [20]).

Although both ORs and IRs are—as odour-gated ion channels—theoretically sufficient to translate the presence of an odour into depolarization of cellular membranes, they operate within complex sensory structures called sensilla (figure 1*a*) [21]. Sensilla are apparent as hair-like projections on the external surface of insects' olfactory organs, the antennae and maxillary palps. Each sensillum overlies a stereotyped combination of OSNs (up to four in *D. melanogaster*), surrounded by various non-neuronal support cells. The ciliated dendrites of OSNs, where olfactory receptors are localized, are housed within the porous shaft of the sensillum and are bathed in lymph fluid. Such organization allows the neuronal sensory membranes to be in close proximity to the odorous environment but protected from physical damage.

Here, we review recent investigations into the development, morphology, biochemistry and physiology of olfactory sensilla, as well as some pertinent examples from similar chemosensory sensilla that mediate taste perception in insects [22]. These advances highlight that the process of chemical detection relies on much more than the receptors alone.

## 2. The morphology and cell biology of olfactory sensilla

Several sensillar types exist (e.g. basiconic, trichoid and coeloconic), which are distinguished by numerous morphological characteristics: length, width, cuticle thickness, and number and size of pores (through which chemicals pass), and neuronal cilia branching complexity (figure 1*a*) [21]. The OSNs in different sensillar classes are often specialized for the detection of particular types of odours; for example, trichoid sensilla neurons are required for pheromone detection, while those in basiconic sensilla mostly detect food-derived odours [9]. This functional relationship, together with the conservation of sensillar types across most insects, suggests that these morphological properties are important for their roles in odour detection.

The sensillar surface represents the first contact point between an odour molecule and the sensory apparatus. As such, early efforts sought detailed descriptions of external sensillar morphology using electron microscopy (EM) [21]. These studies have been extended recently by combining high-resolution atomic force microscopy (AFM) with computational modelling of odour molecule behaviour near the sensillum [23,24]. EM and AFM revealed that the trichoid sensilla of three different moth species—the corn earworm (*Helicoverpa zea*), the bella moth (*Utethesia ornatrix*) and the silk moth (*Bombyx mori*)—are covered with a series of pores and ridges (figure 1*b*) [19,23,24]. In *B. mori*, these morphological data were used to run aerodynamic simulations at the sensillar surface. These analyses suggested that the ridges help to create small vortices that could facilitate the delivery of pheromone molecules into the sensillar pores [24]. Such simulations may help explain surprising observations of early work using radiolabelled pheromones, which estimated that approximately 25% of pheromone molecules adsorbed onto the sensillar surface activate OSNs [25], an efficiency that is greater than 50-fold higher than that predicted by consideration of airflow and pore dimensions alone [24]. Other modelling approaches have considered odour aerodynamics in the context of entire olfactory organs [26], which exhibit substantial morphological diversity across species [27]. For example, many moth antennae comprise arrays of sensilla along multiple, parallel antennal branches, an organization that is likely to maximize the volume of air sifted to detect minute quantities of pheromones [27].

Formation of the sensillar cuticle depends upon the non-neuronal support cells, which secrete the constituent macromolecules, notably chitin and proteinaceous components [21]. Different regions of the hair are formed by distinct types of support cells, from which they get their name: thecogen (sheath cell), trichogen (shaft cell) and tormogen (socket cell) [21]. How the precise sensillar cuticle architecture is determined is largely unknown, but recent work in *D. melanogaster* provided important insights into the formation of the pores in the shaft, at least in maxillary palp basiconic sensilla. Transmission EM revealed that during basiconic sensillum development, the trichogen elongates from the external surface of the epithelium and develops undulations in its plasma membrane where the cuticle envelope layer is secreted [28]. Ultra-thin regions in this envelope that form between protrusions of the plasma membrane correlate with where pores will develop. Screening for genes expressed specifically in the trichogen during development, combined with RNA interference (RNAi)-based functional testing, identified the transmembrane protein Osiris 23/Gore-tex (Osi23) as an important contributor to this process in this sensillar class [28]. Loss of Osi23 led to the disappearance of the plasma membrane undulations, resulting in the formation of a sensillum surface lacking pores; consequently, neuronal responses to odours are dramatically reduced [28]. Osi23 localizes to endosomes, but how it influences plasma membrane morphology is unknown. Interestingly, other members of the Osi family are expressed in cuticle-secreting cells elsewhere in the fly (e.g. those lining the tracheae), hinting at a common role for this insect-specific protein family in shaping cuticular structures [28].

The second key contact point for odours is on the cilia membranes where olfactory receptors are localized. While the construction of OSN cilia and targeting of receptors to this compartment is likely to rely on the conserved intraflagellar transport pathway that is central to the assembly of other types of cilia [29,30], additional potential molecular regulators of these processes have emerged from reverse and forward genetic studies in *D. melanogaster*. For example, inspired by the intimate relationship between cilia function and Hedgehog signalling in vertebrates [31], analysis of OSNs lacking different components of this pathway in flies revealed a contribution to the efficient cilia localization of ORs and robust odour-evoked responses [32]. Unexpectedly, localization of the co-receptor ORCO is apparently insensitive to loss of the Hedgehog pathway. This observation suggests that Hedgehog signalling is required for the assembly of OR/ORCO complexes and/or that ORCO subunits alone can use an independent transport pathway to cilia.

Unbiased genetic screens in *D. melanogaster* revealed a requirement for a lipid transporter homologue, ATP8B, in odour-evoked responses of several OSN classes, including those expressing OR67d, a receptor for the sex and aggregation pheromone 11-*cis* vaccenyl acetate (cVA) [33,34]. ATP8B is expressed and required in OSNs and localized to the ciliated dendrites. The transporter belongs to the P4-type ATPase family, which is thought to flip aminophospholipids (e.g. phosphatidylserine) between membrane leaflets. A predicted enzymatically inactive version of ATP8B fails to rescue the mutant phenotype, while a mammalian homologue can complement the defect, suggesting that the lipid flippase function is critical for its role in OSNs. How lipid composition impacts OR signalling is unclear. One report proposed a role in OR trafficking to cilia, based upon observations of reduced OR67d levels in the cilia of *ATP8B* mutant animals [33]. However, another study saw no defect in OR22a localization upon ATP8B knockout [34]. This discrepancy could reflect differences in the effect of ATP8B function on distinct ORs in different sensillar types, or the inherent difficulty in reliably quantifying protein levels in OSN cilia. It is also possible that lipid composition affects cilia morphology and/or the acute function of these ion channels, as in other biological contexts [35,36].

A significant impediment to relating ultrastructural features of sensilla to molecular components is the difficulty in using standard EM labelling methods. In other tissues, techniques combining EM and genetic labelling have facilitated the integration of morphological and molecular information [37–39]. For example, a diaminobenzadine (DAB)-oxidizing enzyme can be expressed in specific tissues or organelles, where its location is subsequently visualized by staining the oxidized DAB with EM-detectable electron-dense osmium tetraoxide (OsO_4_) [20,40–44]. However, the preservation of tissue ultrastructure during OsO_4_ staining necessitates chemical fixation or cryofixation [45]. Neither of these fixation methods have been easily applied to sensilla because the cuticle is impermeable to chemical fixatives, and cryofixation precludes labelling with DAB-oxidizing enzymes, severely curtailing its utility.

This challenge was recently addressed with the development of the ‘CryoChem' method, in which samples are rehydrated after cryofixation and high-pressure freezing [20]. This treatment preserves sensillar ultrastructure and creates a tissue environment amenable to fluorescent protein and APEX2 (a DAB-labelling protein) function, as well as *en bloc* heavy metal staining (figure 1*c*) [20]. CryoChem has been used in *D. melanogaster* to create three-dimensional reconstructions of several distinct, genetically marked OSNs in different sensilla by serial block-face scanning EM [20,46]. These observations provide useful insights into the relationship between OSN anatomy and olfactory physiology, as discussed below.

Together, these studies emphasize the wealth of cell biological detail that still remains to be discovered in sensilla. Even when proteins essential for sensilla formation are identified by genetic approaches, their mechanism of action can remain unclear [28,47,48]. Further progress requires both continued technical development to visualize the cuticular and membrane ultrastructure of sensilla and mechanistic and developmental characterization of protein function in both OSNs and support cells.

## 3. Non-receptor proteins in olfactory signalling pathways

**Figure 2.**
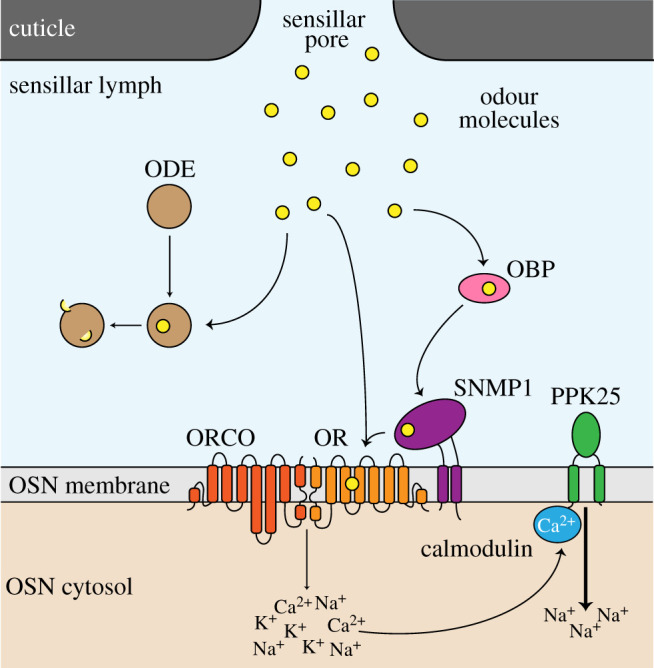
Non-receptor proteins involved in olfactory signalling. Schematic depicting different classes of proteins that act with ORs in pheromone signal transduction (see text for details). The precise path(s) and molecular interactions of pheromone molecules within the sensillum remain unknown.

Olfactory receptors are the central mediators of odour-evoked neuronal responses, often (but not always) sufficient to confer ligand-evoked membrane depolarization in heterologous cell types. However, olfactory signalling in a native context depends upon the interactions of odours with numerous other molecules, as well as the regulation of receptor function (figure 2).

After entering the sensillum, odours must diffuse through the sensillar lymph, an aqueous ionic mixture rich in secreted proteins and proteoglycans [49]. The rate and efficiency with which odours are able to move into the sensillum and through the lymph to reach the OSN membrane is dependent on both the physico-chemical properties of the odours themselves [50,51] and their interactions with proteins in the lymph. Among the lymph proteins, odorant-binding proteins (OBPs) are the most well-studied, although their function remains enigmatic [52]. Members of this family of small, secreted proteins are expressed by sensillar support cells in defined, but often overlapping, sensillar types [53]. *In vitro*, OBPs can bind a multitude of odours with varying degrees of specificity and undergo conformational changes upon ligand binding [52,54]. A historical model for OBP function is that they associate with and transport hydrophobic ligands through the lymph fluid to the OSN; here, they release the odour to the receptors, possibly triggered by local differences in pH near the cilia membranes or through hypothetical interactions of OBPs with cilia membrane proteins [54]. Data from studies on pheromone signalling systems in both *D. melanogaster* and moths is generally consistent with this model, but recent work with other OBPs is not [52], as we discuss below.

In *D. melanogaster,* the OBP LUSH (also known as OBP76a) is required for electrophysiological and behavioural responses to the pheromone cVA [55]. Supraphysiological concentrations of pheromone can evoke some neuronal activity [56], indicating that while LUSH may play a role in delivering cVA to the cognate receptor (OR67d), it is not an integral part of the signal transduction machinery. Recent *in vivo* work in moths has yielded similar results, with genetic knockdown or knockout of pheromone-binding OBPs resulting in 20–60% reductions in global pheromone-evoked antennal electrical activity [57–63] and similar decreases in behavioural responses [57,59,61,63]. The more modest phenotypes observed in moths relative to those in *D. melanogaster* may be due to methodological differences of these studies, but could also reflect functional redundancy between co-expressed moth OBPs [57].

In contrast with pheromone-interacting OBPs, analysis of family members expressed in other sensillar types in *D. melanogaster* has revealed subtler, and sometimes unexpected, roles. Loss of OBP28a in one basiconic sensillum class (ab8) resulted in increased physiological responses to odorants [53], suggesting a role in gain control of odour-evoked activity. However, OBP28a is expressed in several other sensillar classes (which, unlike ab8, express additional abundant OBPs), and responses of these sensilla to other odours were slightly diminished in *Obp28a* mutants [64]. Some OBPs are functionally redundant: simultaneous loss of the co-expressed OBP83a and OBP83b [53] led to delayed deactivation of neuronal responses after odour removal for a subset of OSNs; importantly, this phenotype was rescued by re-expression of either individual protein [65]. In several cases, OBP function has remained elusive: comprehensive expression and mutational analysis of OBPs in six basiconic sensilla classes revealed that simultaneous loss of all proteins within a given sensillum had either no or very minor impact on the responses of OSNs to odour stimuli, which spanned diverse chemical classes, a wide concentration range and varied temporal dynamics [66]. It is possible that these proteins only function in particular biological contexts, as has been suggested for OBP69a, whose expression in pheromone-sensing sensilla is modulated by social interactions of flies [67].

Together, these results indicate that OBPs have diverse, odour-specific and neuron/receptor-specific roles, although the biochemical mechanisms remain unclear in any case. These proteins could be acting as a sink for some odorants, lowering background signals by preventing less ecologically pertinent chemicals from being able to reach receptors. Alternatively, they could clear ligands away after the initial stimulus to preserve the temporal connection between an encounter with an odour and neuronal activity. They could also contribute by binding endogenous lymph molecules: for example, OBP59a is expressed in apparently poreless sensilla in the antenna and is essential for hygrosensory behaviours in *D. melanogaster*, a sensory modality that may not depend upon binding of external molecules [68]. While connecting OBPs' capacity to bind ligands *in vitro* with their physiological and behavioural functions *in vivo* remains a substantial challenge, the appreciation that there may not be a universal function for this protein family may help researchers to maintain an open mind in future explorations.

Other soluble proteins in the sensillar lymph include chemosensory proteins (CSPs) [69,70], Niemann Pick-type C2 (NPC2) homologues [71–76] and odorant-degrading enzymes (ODEs) [77]. CSPs and NPC2 homologues bind myriad small compounds *in vitro* [70,71,73,75], but their *in vivo* functions are almost completely unknown. Some contributions may not necessarily be related to sensory detection: recent work in the malaria mosquito (*Anopheles gambiae*) demonstrates that genetic variants of the leg-enriched CSP SAP2 confer insecticide resistance [78]. These observations suggest that this CSP acts in sequestration/detoxification of environmental chemicals that enter the body through chemosensory sensilla on these appendages.

ODEs are thought to degrade odour molecules in the sensillar lymph [77], which could reduce background neuronal activity and/or regulate odour-evoked temporal dynamics. The first reported ODEs were members of an antennal-specific esterase family [77,79]. Work over the last two decades has discovered other classes of putative ODEs, including some membrane-bound Cytochrome P450s [80–84]. The best-studied ODEs are *D. melanogaster* Esterase 6 and juvenile hormone esterase duplication [85], which evolved from an ancestral juvenile hormone esterase orthologue [86]. These enzymes do not degrade juvenile hormone, but rather break down volatile esters [85,87–89]. Although the exact contributions of these and other ODEs to odour-evoked neuronal responses and behaviour remain unclear [87–90], the loss of the juvenile hormone esterase duplication appears to cause modest decreases in olfactory and behavioural responses to fruit esters [89].

In addition to molecules secreted by support cells, proteins in these cells' membranes may contribute to olfactory signalling. In *D. melanogaster*, the ammonium transporter Amt is thought to be expressed exclusively in the support cells of a coeloconic sensillum class that houses an ammonia-sensing neuron [48]. Genetic analysis revealed that loss of Amt leads to greatly diminished responses to ammonia stimulation [48]. The *A. gambiae* Amt orthologue functions as an ammonia transporter *in vitro* [91], but it is unclear exactly how this activity might contribute to olfactory detection *in vivo*. One hypothesis is that Amt transports ammonia out of the lymph to lower the basal concentration of this chemical near the OSN dendrites, thereby helping to minimize tonic adaptation of the ammonia-sensing neuron [48]. However, a recent analysis of *A. gambiae Amt* using transgenic tools indicated that this gene is expressed in both support cells and OSNs [92], raising questions about its cellular site(s) of action. Regardless of the precise mechanism, Amt represents an interesting case where an integral membrane transporter can directly affect olfactory detection. Many other uncharacterized putative transporter proteins are expressed in the antenna [48], and it will be interesting to determine whether any of these have analogous roles.

Non-receptor proteins in the OSN cilia membrane can also play important roles in olfactory transduction. The best characterized is Sensory Neuron Membrane Protein 1 (SNMP1), a two-pass transmembrane protein related to the mammalian CD36 family [93]. SNMP1 was originally characterized in moths for its expression and ciliary localization in pheromone-sensing neurons [94], properties that are broadly conserved in insects [95–98]. Mutational analysis in *D. melanogaster* demonstrated that SNMP1 is essential for OR67d-mediated responses to cVA [95,99]. However, the requirement for SNMP1—like that of the OBP LUSH—can be bypassed by very high concentrations of this pheromone [100], indicating it is not a strictly essential part of the receptor complex. The important role of SNMP1 in pheromone detection in *D. melanogaster* is likely to be conserved in other insects. For example, RNAi of *Snmp1* in *B. mori* impairs the ability of males to locate and mate with females, processes that depend heavily upon pheromone detection [98]. Mammalian CD36 proteins bind/transport lipid-like molecules in diverse cellular contexts, and the partial ability of a murine CD36 homologue to rescue *Snmp1* mutants in *D. melanogaster* [97] has guided mechanistic studies of SNMP1 function. A homology model of SNMP1, based on the crystal structure of the CD36 protein LIMP-2, predicts that the SNMP1 ectodomain has a hydrophobic cavity [97], which may act as a conduit for transporting hydrophobic pheromone molecules from the extracellular lymph to a closely apposed OR complex in the cilia membrane [95,97,101,102]. While the mechanism remains to be fully established, the critical requirement for OBPs and SNMP1s in the detection of pheromones, but not other types of odours, may be related to the biochemical challenges of concentrating generally large and highly hydrophobic pheromone ligands at the surface of the OSN membranes.

Olfactory neuron responses can be further modulated by other signalling molecules after receptor activation. A longstanding question is how insect ionotropic olfactory receptors attain sufficient sensitivity without signal amplification by second messengers, which is inherent to vertebrate metabotropic chemosensory receptor transduction [103]. Recent work offers a solution to this problem by providing evidence that the degenerin/epithelial sodium channel Pickpocket 25 (PPK25) amplifies ligand-evoked currents downstream of certain olfactory receptors [104]. In *D. melanogaster*, the genetic knockout or overexpression of PPK25 decreases or increases, respectively, the physiological sensitivity of Or47b OSNs [104], a class of pheromone-sensing neurons involved in courtship behaviour [105,106]. These effects are dependent on calmodulin, as a mutation of a calmodulin-binding motif in PPK25 or pharmacological inhibition of calmodulin mimics loss of PPK25 [104]. Interestingly, this role of PPK25 can also be observed for OSNs expressing IR84a, which recognizes food-derived odours that promote courtship behaviour [107], and for a population of gustatory sensory neurons (GSNs) that detects non-volatile pheromones [104]. The revelation that this PPK acts as a signal amplifier, rather than a sensory receptor, in these different classes of neurons has potentially broad significance: many members of the *D. melanogaster* PPK family have been implicated in diverse sensory modalities but it has been unclear (with one exception [108,109]) whether they are the sensory receptors or not [110–124]. These channels may have analogous roles beyond sensory systems; for example, PPK11 and PPK16 modulate presynaptic membrane voltage at the neuromuscular junction to regulate homeostatic plasticity [125].

Some olfactory receptor subunits may also act as modulators of receptor activity, rather than binding ligands themselves. For example, several IR-expressing OSNs express—in addition to a tuning receptor and co-receptor—a third receptor protein, IR76b [13]. Some evidence points to IR76b acting as a critical component of a putative tripartite olfactory receptor complex [17,126], which is also concordant with the broad expression and function of this protein in various GSN populations [126–132]. However, a distinct role for IR76b in limiting, rather than contributing to, ligand-evoked responses has emerged through analysis of a population of GSNs that detect both sugars and acetic acid. Here, the mutation of IR76b leads to increased ligand-evoked physiological responses, with a corresponding enhancement of behavioural sensitivity [133]. The function of IR76b as a dampener of neuronal responses exhibits some specificity, as the sensitivity of a mammalian capsaicin receptor that is ectopically expressed in these sugar/acid-sensing neurons is not affected in *Ir76b* mutants. Moreover, ectopic IR76b expression in other neuronal populations does not reduce their physiological responsiveness [133]. The context-dependent modulatory role of IR76b is reminiscent of mosquito carbon dioxide receptors, which comprise two subunits that are essential for ligand-evoked responses, and a third that may modulate ligand-evoked sensitivity [134–136]. The mechanistic basis of receptor subunit modulation is unknown in any case, but these findings highlight the intricate regulation that may occur between subunits within (putative) heteromeric complexes to shape ligand-dependent ion conduction.

## 4. Biophysical properties and intercellular regulation of olfactory sensory neuron responses

**Figure 3.**
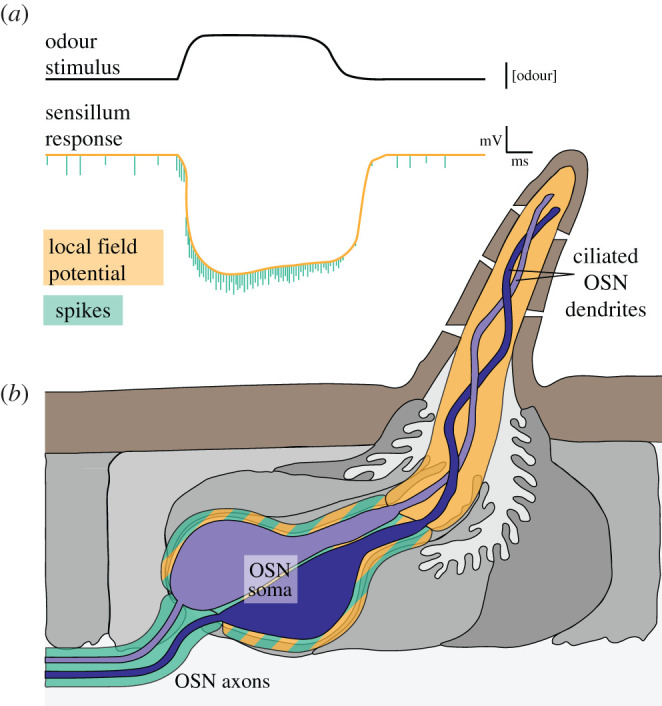
Peripheral olfactory physiological processes. (*a*) An idealized drawing of a sensillar olfactory response, illustrating the two main physiological processes. (*b*) Schematic of a sensillum depicting regions where these physiological responses occur. Although spikes are thought to be generated in the OSN axons, they can be detected experimentally in the dendrites in the sensillum shaft, possibly through backpropagation.

OSN signalling consists of two physiological processes: first, odour-dependent gating of the olfactory receptor channel, ion flow and cilia membrane depolarization, and second, conversion and propagation of this initial signal by voltage-gated channels in the form of action potentials (or spikes) down the OSN axon [137–139] (figure 3). The first of these processes can be detected as changes in a sensillum's local field potential (LFP), representing the transient electrical potentials in the sensillum generated by OSNs, as well as contributions from the ion transport activities of support cells [138,139]. LFP dynamics reflect signal transduction properties that are determined by the specific nature of odour ligand/receptor interactions, while the temporal dynamics of spiking can be described by a linear filter that is stereotyped across different OSN classes [138,140].

Most olfactory physiological studies do not measure LFP and use spike frequency as the sole proxy for reporting odour-evoked neuronal activity [138,141,142]. While spikes represent the information that is transmitted to the brain, a comprehensive appreciation of peripheral OSN physiology is crucial to understand responses to naturalistic odour stimuli. Odours exist as plumes comprising pockets of air containing wide-ranging concentrations of chemicals. OSNs respond to this temporally complex stimulus pattern in diverse ways, such as desensitization to strong stimuli, or sensitization to repeated weak stimuli [137,138,140,143,144]. LFP and spike rate exhibit very different adaptation kinetics [138,140] and also appear to adapt in response to different aspects of the odour stimulus. For example, LFP, but not spike rate, adapts strongly in response to changes in the mean stimulus intensity [140]. By contrast, both LFP and spike rate are influenced by the variance in an odour stimulus, although the adaptation dynamics of each component differs [140]. LFP and neuron spiking are, of course, intimately connected phenomena, and while the kinetics of spike rate and LFP are distinct, the dynamics of changes in spike amplitude are nearly identical to those of LFP [145]. Together, these analyses reveal the sophistication with which OSNs encode different aspects of odour stimuli and emphasize that measurement of spike frequency alone does not fully capture OSN responsiveness and therefore our ability to understand how odour-evoked neuron activity arises.

The molecular basis of the dynamic physiological properties of OSNs remains unclear. Most analyses have focused on structure/activity dissection of ORCO, providing evidence that sensory adaptation relies upon modulation of both receptor localization and sensitivity. This co-receptor (and, presumably, its partner tuning OR) were observed to be depleted from cilia upon prolonged odour exposure, although this was measured only over a multi-day time scale [146]. Activity-dependent control of ORCO localization may rely on calmodulin: RNAi of calmodulin or mutation of a predicted calmodulin-binding motif in ORCO's second intracellular loop disrupts its cilia localization, with consequent defects in odour-evoked activity [146]. Physiological studies have provided additional evidence for the role of calcium signalling and/or calmodulin in ORCO-dependent sensitization of neurons to repeated odour stimulation [147] and sensory adaptation of OR-expressing neurons [148]. The same loop of ORCO also contains three potential phosphorylation sites [144,149,150]. Mutation of these sites reduces ORCO's conduction properties in heterologous cells [149] and diminishes OSN sensitivity and behavioural responses to odours *in vivo* [150]. In addition, mutation of ORCO's phosphorylation sites prevents odour sensitization [143]. One of these sites, S289, is dephosphorylated *in vivo* upon OSN desensitization [144,151]. An ORCO^S289A^ mutation reduces OSN sensitivity *in vivo*, and a phospho-mimetic mutant (ORCO^S289D^) reduces the magnitude of OSN desensitization after odour exposure [144]. These studies begin to unveil the complexity of olfactory receptor regulation that contributes to the temporal response properties of OSNs, but also make apparent the challenge of cleanly dissecting effects on receptor localization and/or activity.

The molecular regulation of other types of olfactory receptors is essentially unknown, although N-glycosylation has been implicated in the control of IR localization and activity [152]. Electrophysiological studies indicate that Or and Ir neurons have distinct temporal response properties [148,153], at least some of which appear to be dependent on the receptors themselves [153]. Moreover, the acute contribution of pathways implicated in sensillar development, such as Hedgehog signalling or the lipid flippase ATP8B (discussed above), remains to be explored.

Beyond autonomous regulatory mechanisms in OSNs, recent work has characterized the interdependence of the activity of different OSNs within the same sensillum. In many sensillar classes, the activity of one OSN is inhibited upon activation of a neighbouring neuron [154]. Blocking synaptic transmission does not prevent such inhibitory interactions between the two OSNs [154], nor is there evidence for gap junctions between paired OSNs. These observations suggest that the inhibition occurs through ephaptic coupling [46,154], a phenomenon in which the activity of one neuron alters the local electric field to impair depolarization of a nearby neuron. In support of this hypothesis, simultaneous recording of two different sensilla that are artificially coupled by a metal electrode demonstrated that continuous stimulation of a neuron in one sensillum can be inhibited by excitation in the adjacent sensillum [46]. Moreover, the combination of these observations with EM analysis of defined neuron types via CryoChem (described above) revealed that the inhibitory effect is stronger when exerted from a larger OSN onto a smaller OSN [46]. A plausible explanation for this asymmetric relationship is that bigger neurons are expected to have lower input resistance and a greater dendritic surface area to allow for a higher maximal LFP [46], hinting at a previously unappreciated link between OSN morphology and physiology. Future work will determine how such ephaptic interactions impact odour coding, in particular of complex natural odour blends.

Work on GSNs in the bumblebee (*Bombus terrestris*) provides interesting additional insights into how, and why, neurons within the same sensillum communicate [155]. Recordings from a highly sensitive sugar sensing neuron in ‘type A' sensilla on the mouthparts revealed an unusual bursting pattern of spikes upon stimulation with high concentrations of sucrose [155]. The end of the spike burst coincides with a single spike from a second neuron in this sensillum. Introduction of a gap junction inhibitor into the sensillum led to the continuous sucrose-evoked firing of the first neuron, suggesting that—in contrast with the ephaptic inhibition described in olfactory sensilla—the second neuron terminates the first neuron's spike train via electrical synapses (these structures have not, however, been visualized directly). Importantly, this bursting pattern of firing prevents neuronal desensitization, which may explain the ability of bees to sustain feeding behaviour on high-sugar nectar [155].

## 5. Conclusion and perspectives

The discovery of insect olfactory receptors has been instrumental in understanding how these animals detect environmental odours, as well as facilitating the development of molecular tools to map and manipulate olfactory circuits. However, receptors alone do not define the exquisite sensitivity, specificity and temporal precision observed in odour-evoked neuronal activity. We have highlighted the complexity of peripheral signal transduction in olfactory sensilla, and the extraordinary wealth of biology that remains to be uncovered. It is clear that many neuronal, non-neuronal and secreted molecules that participate in this process (or rather processes) have still to be characterized [156]. Moreover, determination of the *in vivo* function of most proteins in defining signalling properties, and how these impact behavioural responses, will require technical innovations to permit their acute inhibition to distinguish roles in sensillar development from direct contributions to signal transduction. Finally, while investigating insect olfactory transduction is of widespread interest in sensory neuroscience and chemical ecology, many of the insights gained are likely to have broad relevance for understanding molecular and cellular communication processes across diverse tissues and species.

## Data accessibility

This article has no additional data.
